# Jasmonic Acid and Ethylene Participate in the Gibberellin-Induced Ovule Programmed Cell Death Process in Seedless Pear ‘1913’ (*Pyrus hybrid*)

**DOI:** 10.3390/ijms22189844

**Published:** 2021-09-11

**Authors:** Huibin Wang, Shichao Zhang, Yingying Qu, Rui Gao, Yuxiong Xiao, Zhigang Wang, Rui Zhai, Chengquan Yang, Lingfei Xu

**Affiliations:** 1 College of Horticulture, Northwest A&F University, Taicheng Road. 3, Yangling, Xianyang 712100, China; wanghuibin069@nwsuaf.edu.cn (H.W.); woshizsc@nwsuaf.edu.cn (S.Z.); 2019050262@nwsuaf.edu.cn (Y.Q.); hopppie@nwsuaf.edu.cn (R.G.); yuxiongx@nwsuaf.edu.cn (Y.X.); Zhai.Rui@nwsuaf.edu.cn (R.Z.); cqyang@nwsuaf.edu.cn (C.Y.); 2 State Key Laboratory of Crop Stress Biology for Arid Areas, Northwest A&F University, Taicheng Road, Yangling, Xianyang 712100, China

**Keywords:** seedless pear, GA, JA, SA, ethylene, ovule, PCD, *PbSAG39*

## Abstract

Seedless fruit is a feature appreciated by consumers. The ovule abortion process is highly orchestrated and controlled by numerous environmental and endogenous signals. However, the mechanisms underlying ovule abortion in pear remain obscure. Here, we found that gibberellins (GAs) have diverse functions during ovules development between seedless pear ‘1913’ and seeded pear, and that GA_4+7_ activates a potential programmed cell death process in ‘1913’ ovules. After hormone analyses, strong correlations were determined among jasmonic acid (JA), ethylene and salicylic acid (SA) in seedless and seeded cultivars, and GA_4+7_ treatments altered the hormone accumulation levels in ovules, resulting in significant correlations between GA and both JA and ethylene. Additionally, SA contributed to ovule abortion in ‘1913’. Exogenously supplying JA, SA or the ethylene precursor 1-aminocyclopropane-1-carboxylic acid promoted ‘Bartlett’ seed death. The regulatory mechanism in which ethylene controls ovule death has been demonstrated; therefore, JA’s role in regulating ‘1913’ ovule abortion was investigated. A further study identified that the JA signaling receptor MYC2 bound the *SENESCENCE-ASSOCIATED 39* promoter and triggered its expression to regulate ovule abortion. Thus, we established ovule abortion-related relationships between GA and the hormones JA, ethylene and SA, and we determined their synergistic functions in regulating ovule death.

## 1. Introduction

Pear as an important fruit, both nutritionally and commercially, and is planted worldwide. Presently, seedless fruit is desired by consumers, and various seedless species, including grapes and pears, have been bred in the past several years [1,2,3]. The fresh fruit of seedless pear has a high nutritional quality and is more convenient to consume. Thus, identifying the genetic mechanisms and key regulatory genes involved in seedless fruit production is important for pear breeding and meeting market demand. In seeded plants, abnormal fertilization, female and male sterility, embryo abortion, hormone regulation and various other factors cause seedlessness [4]. In a previous study, we identified the seedless pear variety ‘1913’, in which seedlessness is caused by failed fertilization. After pollination, a potential programmed cell death (PCD) mechanism in ovules is triggered in ‘1913’ [4]. Although seed abortion has been studied, there are still many unknown details.

As a basic cellular process, PCD has adopted a series of vital functions in multicellular organisms. In individual organisms, the gene coding procedures associated with aging and death control generational replacement, which is the driving force of adaptive evolution. In plants, there are various forms of PCD, and they are important in development and in abiotic and biotic stress responses. During reproductive and vegetative development, development-controlled PCD (dPCD) occurs and plays key roles. It often ends senescence or the functions of no longer required cells [5]. Additionally, different dPCD events are distinguishable on the basis of the developmental context. Senescence is best-defined as the process of growing old. In plant biology, senescence is often used to describe the reproducible patterns of cell death and biochemical changes, and it is considered to be a type of PCD [6].

In plants, various hormones participate in PCD processes. For instance, ethylene, jasmonic acid (JA) and salicylic acid (SA) have been implicated in PCD signaling [7,8,9,10]. Among them, ethylene is the best-characterized dPCD hormone. Ethylene has been described as an important regulator in controlling the early PCD process in plants, for example, ethylene signaling is required for synergid degeneration [11], nucellus PCD [12], endosperm development [13] and ovule senescence [14]. Additionally, ETHYLENE-INCENSITIVE 3-LIKE (EIL1), the receptor of ethylene signaling, directly activates the expression of the senescence-related gene *CYSTEINE PROTEINASE 1* (*Cysp1*) to regulate ovule senescence in pear [4]. Another important hormone in plant development, JA, is essential for plant defense and development, and it serves as an important signal that triggers senescence-associated gene expression and tissue senescence [8]. MYELOCYTOMATOSIS (MYC) transcription factors (MYC2, 3 and 4) function as the general switches in the JA-signaling pathway, and they negatively regulate cell cycle-related gene expression and inhibit plant growth [15,16]. In *Arabidopsis*, MYC2 directly binds the promoter and activates the expression of *SENECENCE-ASSOCIATED GENE 29* (*SAG29*) to trigger JA-induced leaf senescence [8]. However, there are limited reports on the relationship between JA and ovule senescence. In plants, ovules are derived from specialized meristematic regions within the carpels, referred to as the placentas, and carpels are proposed to have evolved from ancestral foliar organs, either leaf-like sporophylls that folded to enclose the ovules or bract-like structures that subtend shoot-like ovules [17]. Additionally, a gene homologous to the *Arabidopsis* leaf senescence-associated gene *SAG12* in pear, *SAG39* (LOC103945424), is expressed in ovules [4]. Thus, there may be similar regulatory mechanisms between leaf and ovule senescence. In plants, SA, which has been widely studied in disease resistance, including pathogen-mediated response and cell death, may be involved in senescence-associated cell death [18]. Evidence indicates that SA is also associated with leaf senescence. When leaves are undergoing senescence, there is a high SA level, and the *npr1* and *pad4* mutants, which are defective in SA signaling, show reduced expression levels of a number of *SAG* genes, including *SAG12* in *Arabidopsis* [19]. Additionally, SA’s regulation of gene expression during developmental senescence has been reported by Buchanan-Wollaston et al. [20].

Hormones function as important mediators in the regulation of seed development and abortion. In the enlightenment period of seed development, the biosynthesis of various hormones, such as auxin and gibberellin (GA), is activated, and hormones appear to have prominent roles in synchronizing fertilization and ovule growth [21]. In *Arabidopsis*, pollination triggers the biosynthesis of auxin in ovules, and then auxin signaling promotes active GA biosynthesis in ovules, which promotes ovule development [21]. As an important hormone, GA plays fundamental roles in various plant developmental processes, including ovule development. The ectopic expression of pea GA inactivates the enzyme 2-oxidase in *Arabidopsis*, causing seed abortion [22]. In pea, mutations of the GA biosynthesis genes *LH1* and *LH2* greatly affect embryogenesis and cause the production of abnormal seeds [23]. Thus, GAs appear to positively regulate ovule development at early stages. Therefore, determining whether GA applications could block seed abortion in seedless pear is of interest.

In this study, we found that GA played different roles in the ovule development of seeded and seedless pear. A GA_4+7_ treatment triggered a potential PCD process in seedless pear, and we determined that JA and ethylene participate in GA-induced ovule abortion. In addition, SA is involved in pear ovule abortion, although it did not respond to the GA_4+7_ treatment. Furthermore, a link between JA signaling and pear ovule abortion was established, in which PbMYC2 directly mediates the expression of *PbSAG39*, leading to ovule abortion. These results provide a meaningful foundation for understanding ovule abortion and are useful for seedless fruit breeding.

## 2. Results

### 2.1. GA_4+7_ Advances the PCD Process of ‘1913′ Unpollinated Ovules

**Figure 1:**
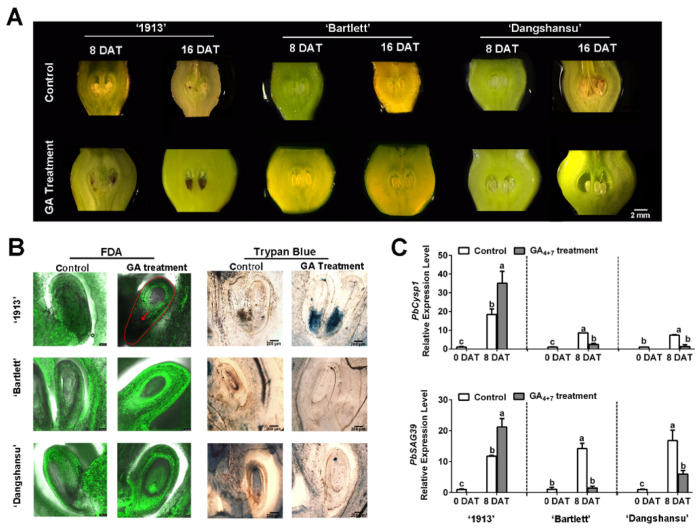
Ovule development of samples taken at 8 and 16 days after treatment (DAT) from ‘1913’, ‘Bartlett’ and ‘Dangshansu’. (**A**) Observations of ovule development at 8 and 16 DAT. (**B**) The ovules of 8 DAT from ‘1913’, ‘Bartlett’ and ‘Dangshansu’ were stained by FDA and trypan blue. (**C**) Expression profile of ovule senescence-related genes *PbCysp1* and *PbSAG39* in ovules of 0 and 8 DAT from ‘1913’, ‘Bartlett’ and ‘Dangshansu’. The sample of 0 DAT means the sample was collected before treatment. Control: unpollinated ovules with solvent treatment. GA Treatment: GA_4+7_ treatment. The data are mean ± SD of three biological replicates. Error bars represent the standard deviation. Different lowercase letters indicate significant difference at *p* < 0.05 (Duncan’s multiple range test).

To identify the relationship between GA and seedless pear ovule abortion, we sprayed GA_4+7_ on unpollinated ovaries of ‘1913’, ‘Bartlett’ and ‘Dangshansu’ at the full-bloom stage. The morphological observations revealed that GA_4+7_-treated ‘1913’ ovules began to brown at 8 days after treatment (DAT), and the whole ovule turned completely brown or black by 16 DAT. On the contrary, the GA_4+7_-treated ovules of seeded pears ‘Bartlett’ and ‘Dangshansu’ developed normally until 16 DAT (Figure 1A). However, the solvent-treated ovules of the three pear varieties were normal at 8 DAT, and small areas on the ovules began to brown by 16 DAT (Figure 1A). 

Then, ovule cell development was observed at 8 DAT using FDA and trypan blue-staining assays. There were no fluorescence signals, and obvious blue staining was observed at the adaxial-funiculi of ‘1913’ GA_4+7_-treated ovules, whereas the other treated ovules had obvious fluorescence signals but were not stained by trypan blue (Figure 1B). These observations indicate that the ‘1913’ ovules were dead at 8 DAT with GA_4+7_. To further investigate, we analyzed the expression profiles of *PbCysp1* and *PbSAG39*, which are genes in pear homologous to *Cysp*1 in *Brassica napus* and *SAG12* in *Arabidopsis*, respectively. *Cysp1* and *SAG12* are marker genes of PCD and participate in ovule senescence [14,24]. The qRT-PCR indicated that GA_4+7_ increased the expression levels of *PbCysp1* and *PbSAG39* in ‘1913’ ovules at 8 DAT, but it reduced their expression levels in ‘Bartlett’ and ‘Dangshansu’ ovules, compared with the solvent-treated ovules (Figure 1C). In comparison to the expression levels of the two marker genes in solvent-treated ovules at 0 DAT, the levels significantly increased in the three pear varieties by 8 DAT, which suggested that the ovules underwent a PCD process when not pollinated at the full-bloom stage. Additionally, GA_4+7_ appeared to promote the PCD process in ‘1913’ ovules and inhibit the PCD process in ‘Bartlett’ and ‘Dangshansu’, which is consistent with the morphological observations and staining assays. Thus, GA_4+7_ may trigger a potential PCD process in the seedless ‘1913’ pear variety.

### 2.2. SA, JA and Ethylene Enriched in ‘1913′ Ovules 

**Figure 2:**
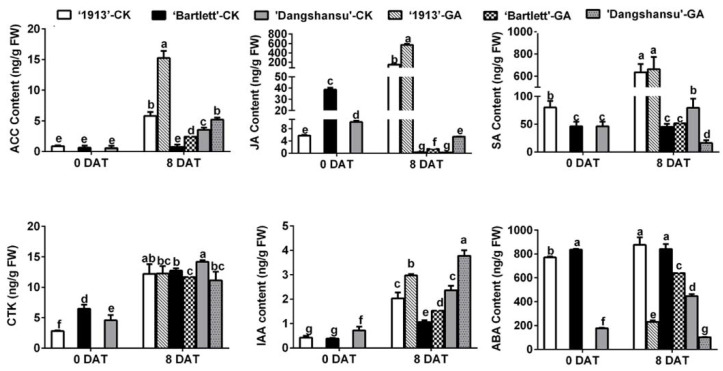
Hormone content of ovules at 0 and 8 DAT from ‘1913’, ‘Bartlett’ and ‘Dangshansu’, including ACC (aminocyclopropane-1-carboxylic acid, the precursor of ethylene), JA, SA, CTK (cytikinins), IAA and ABA. The samples at 8 DAT were collected from ovules with GA_4+7_ treatment and untreated. DAT, days after treatment. The data are mean ± SD of three biological replicates. Error bars represent the standard deviation. Different lowercase letters indicate significant difference at *p* < 0.05 (Duncan’s multiple range test).

To determine why the GA_4+7_ treatment had different effects on ovule development between seedless and seeded pears, endogenous hormones were quantified in the ovules of ‘1913’, ‘Bartlett’ and ‘Dangshansu’ at 0 and 8 DAT. By analyzing the solvent-treated ovules in the three pear varieties at 8 DAT, the results showed that the levels of 1-aminocyclopropane-1-carboxylic acid (ACC), which is the precursor of ethylene, JA and SA were significantly different in seeded and seedless ovules at 8 DAT, and their concentrations were greater in the ovules of ‘1913’ than in ‘Bartlett’ and ‘Dangshansu’. A similar difference was also observed in GA_4+7_-treated ovules, but in contrast, the contents of CTK, IAA and ABA did not have absolutely consistent differences between seeded and seedless ovules (Figure 2). Moreover, GA_4+7_ treatments increased the content of ACC, JA and IAA in three pear ovules at 8 DAT, and ABA content was reduced by GA_4+7_ treatments, compared with the solvent-treated ovules (Figure 2). Furthermore, by comparing the hormone content in solvent-treated ovules between 0 and 8 DAT, we found that the upregulated levels of ACC, JA and SA in ‘1913’ ovules by 8 DAT were more significant than those in seeded pear ovules (Figure 2). These results suggest that ethylene, JA and SA may be involved in the death of ‘1913’ ovules and the increased level of ethylene and JA by GA_4+7_ treatment may contribute to the GA_4+7_-induced ‘1913’ ovule abortion.

### 2.3. GA_4+7_ Treatment Increases the Expression Levels of JA and Ethylene Pathway-Related Genes

**Figure 3:**
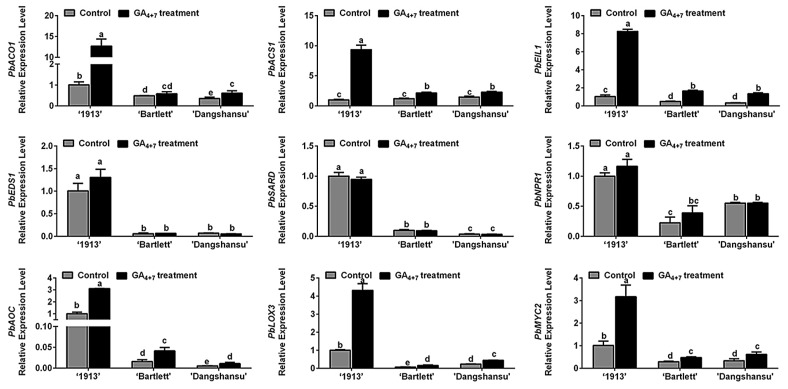
Relationship analyses between ethylene, JA and SA with GA_4+7_ treatment. The expression profile analyses of ethylene, JA, SA synthesis and signaling response-related genes between GA_4+7_-treated and untreated ovules at 8 DAT. The data are mean ± SD of three biological replicates. Error bars represent the standard deviation. Different lowercase letters indicate significant difference at *p* < 0.05 (Duncan’s multiple range test).

Ethylene, JA and SA may participate in ‘1913’ ovule death, and in order to determine the relationship between candidate hormones ethylene, SA, JA and GA_4+7_ treatment, the expression of their biosynthesis and signaling response-related genes were assessed by qRT-PCR. Comparing the solvent treatment (control) and GA_4+7_ treatment groups at 8 DAT, we found that GA_4+7_ treatment increased the expression levels of *PbACO1*, *PbACS1*, *PbEIL1*, *PbAOC1*, *PbLOX3* and *PbMYC2*, and the differences in ‘1913’ were more significant than those in ‘Bartlett’ and ‘Dangshansu’ (Figure 3). Consistent with the hormone level changes (Figure 2), there were no differences in the expression levels of SA synthesis genes (*PbEDS1* and *PbSARD*) and a signaling response-related gene (*PbNPR1*) between GA_4+7_-treated and control groups (Figure 3). These data collectively indicate that the GA_4+7_ treatment promoted the biosynthesis and signaling transduction of ethylene and JA, which accelerated the ovule death process in ‘1913’.

### 2.4. Differences in Ethylene and JA Pathway Gene Enrichments Result in Different Cell Death-Gradient Orientations between ‘1913′ and Seeded Pears

**Figure 4:**
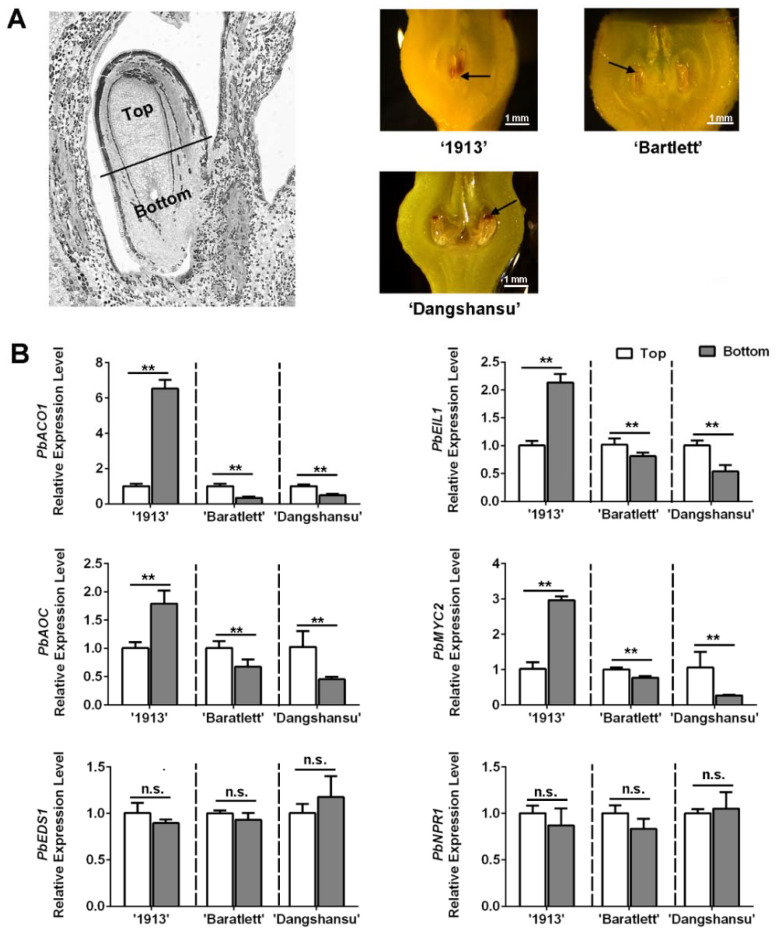
The distribution of JA, SA and ethylene in ovules of seedless pear ‘1913’ and seeded pear ‘Bartlett’ and ‘Dangshansu’ is different. (**A**) The left pear ovule is divided into two parts, the top (the abaxial-funiculus tissue) and bottom (the adaxial-funiculus tissue). Right exhibits that the unpollinated ovules of ‘1913’, ‘Bartlett’ and ‘Dangshansu’ at 16 DAT show different locations of ovule cell death. The black arrows point to areas of significant ovule cell death. (**B**) The expression level of ethylene, JA and SA pathway-related genes on the top and bottom ovule tissue from the seeded and seedless pear. The samples were collected from unpollinated ovules at 16 DAT. The expression level of each gene in the top sample was normalized to 1.0. The data are mean ± SD of three biological replicates. Error bars represent the standard deviation. Asterisks indicate significant difference at ** *p* < 0.01 (Student’s *t*-test). n.s., no significance.

Observations of unpollinated ovule development at 16 DAT revealed a difference between seedless and seeded pear. We collected the unpollinated ovules with solvent treatment and found that ovule death in ‘1913’ fist occurred at the bottom, whereas in ‘Bartlett’ and ‘Dangshansu’ pear, it occurred at the top (Figure 4A). The ovules were then divided into top and bottom samples. An analysis of the expression levels of ethylene, JA and SA pathway genes in ‘1913’ revealed that the levels of *PbACO1*, *PbEIL1*, *PbAOC* and *PbMYC2* were higher at the bottoms of ovules than at that the tops (Figure 4B). After analyzing the ethylene- and JA-related genes in ‘Bartlett’ and ‘Dangshansu’, we confirmed that their expression differences, between the tops and bottoms of ovules, were opposite to those in ‘1913’ ovules (Figure 4B). The samples consisted of solvent-treated ovules at 16 DAT from ‘1913’, ‘Bartlett’ and ‘Dangshansu’. In addition, there were no significant differences in the expression gradients of SA-related genes, including *PbEDS1* and *PbNPR1* (Figure 4B). The data further suggest that ethylene and JA are involved in the death of seedless pear ovules.

### 2.5. Ethylene, JA and SA Treatments Increased the Expression of PCD-Related Genes

**Figure 5:**
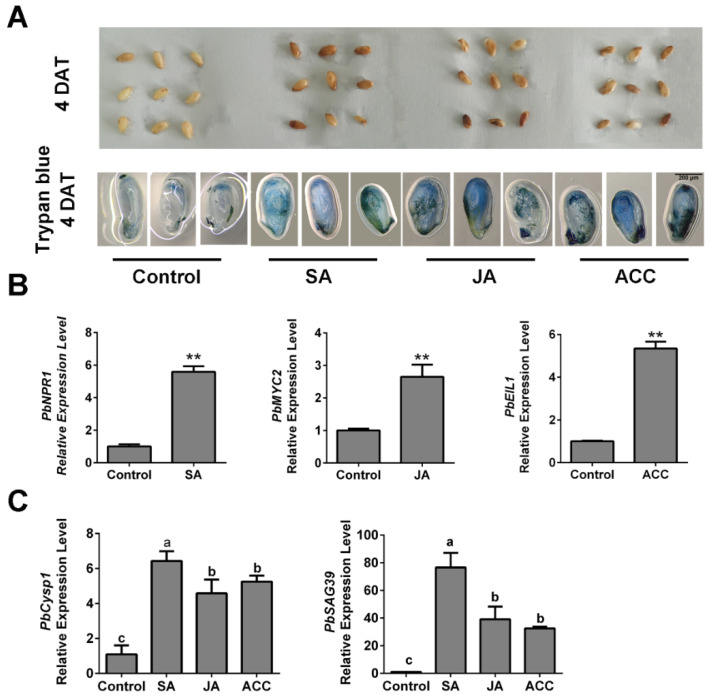
In vitro assay, application of ACC, SA and JA promotes normal seed death. (**A**) Observation of the seed development after treating with ACC, SA and JA respectively, and trypan blue staining assay demonstrated the cell death of different treated ovules. (**B**) Expression level analysis of the SA, JA and ethylene signaling response-related genes. (**C**) The expression of senescence-related genes *PbCysp1* and *PbSAG39* verified the seed death. The data are mean ± SD of three biological replicates. Error bars represent the standard deviation. Asterisks indicate significant difference at ** *p* < 0.01 (Student’s *t*-test). Different lowercase letters indicate significant difference at *p* < 0.05 (Duncan’s multiple range test).

To further determine the relationships between ethylene, JA and SA levels and ovule death in pear, we generated high-hormone level samples by spraying ACC, JA and SA independently on ‘Bartlett’ pollinated ovules in vitro. At 4 DAT, the ACC-, JA- and SA-treated ovules showed obvious browning on their surfaces, compared with the control (Figure 5A). Using trypan blue staining, we observed that obvious tissue death occurred in the hormone-treated ovules (Figure 5A), which indicated that each of the three hormone treatments increased ovule death. Then, we analyzed the *PbNPR1*, *PbMYC2* and *PbEIL1* expression levels independently in SA-, JA- and ACC-treated samples. Their increased expression levels indicated that the three hormones function in ovules (Figure 5B). In addition, the individual SA, JA and ACC treatments increased the expression levels of senescence-associated genes in the ovules (Figure 5C). These results suggest that SA, JA and ethylene affect pear ovule PCD by increasing the expression levels of the cell death-related genes. Thus, we confirmed that ethylene and JA participate in the GA-induced advanced abortion of ‘1913’ ovules and that SA may also be involved, although it did not respond to the GA_4+7_ treatment.

### 2.6. PbMYC2 Directly Triggers the Expression of PbSAG39

**Figure 6:**
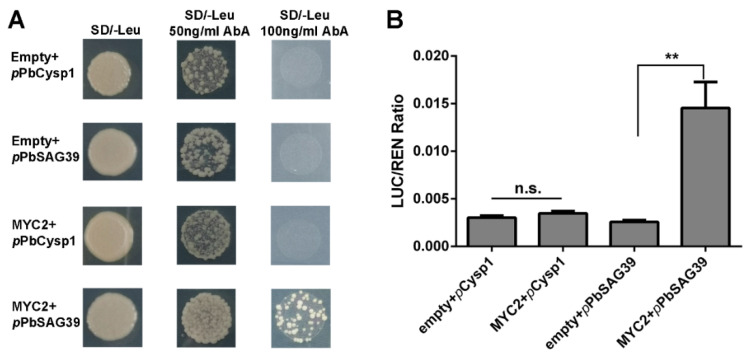
PbMYC2 promotion of PbSAG39 expression by direct binding to the *PbSAG39* promoter. (**A**) Yeast one-hybrid assay verified the interaction of PbMYC2 with *PbCysp1* and *PbSAG39*. (**B**) Validation of the activation effect of PbMYC2 on the *PbSAG39* promoter based on a dual-luciferase assay. Relative promoter activity is represented by the ratio of the activity of the structural gene luciferase (LUC) to that of 35S Renilla (REN). An empty vector was used as a reference. Asterisks indicate significantly elevated LUC activity compared with that in the negative control (** *p* < 0.01, Student’s *t*-test). n.s., no significance.

In this study, JA and ethylene were found to be associated with the GA-triggered PCD process in the seedless pear ‘1913’. Since the regulatory mechanism in which ethylene controls pear ovule PCD was elucidated in our previous study [4], it is clear that the JA-related mechanism controlling leaf senescence acts through MYC factors [8]. Consequently, we further investigated how JA participates in the PCD process of seedless pear ovules. MYC2 acts as a positive regulator of JA signaling to control downstream development. An analysis of the promoter sequences of ovule senescence-related genes *PbCysp1* and *PbSAG39* revealed typical G-box sequences in the promotors, which are MYC-binding sites. Expression profile analyses suggested that there were positive correlations among *PbMYC2*, *PbCysp1* and *PbSAG39* (Figure 5B,C). Using Y1H and dual-luciferase assays, we demonstrated that MYC2 directly bound the promoter of *PbSAG39* and enhanced its expression, but this was not true for *PbCysp1* (Figure 6). This suggests that JA participated in ‘1913’ ovule death through MYC2’s direct triggering of *PbSGA39* expression, which leads to ovule death.

## 3. Discussion

At the early stages of ovule development, the biosynthesis of various hormones, such as auxin and GA, is activated, and hormones play prominent roles in synchronizing fertilization and ovule growth [21]. In this study, we determined the roles of GA, JA, SA and ethylene in the control of ovule development and their relationships during ovule ontogenesis in seedless and seeded pear.

### 3.1. Ovule Death Was Advanced in ‘1913′ Pear by a GA_4+7_ Treatment through Increased JA and Ethylene Levels

Hormones play important roles in plants in the early developmental stages [25]. In plants, GAs are plant growth regulators controlling various aspects of plant growth and development [26]. A previous study reported that fertilization breaks the restrictions of ovule and fruit development in *Arabidopsis*, and during this process, it triggers the biosynthesis of GA in the ovules, which regulates ovule development [21]. This suggests that GAs play essential roles in ovule development, and evidence indicates that GAs potentially contribute to seed development. In this study, we found that ovule death was advanced in the seedless pear ‘1913’ variety, but death was prevented in seeded pears after GA_4+7_ treatments, compared with solvent-treated ovules (Figure 1). We previously analyzed the death of ‘1913’ ovules after pollination and determined that at 7 days after pollination, ovule death occurred, and cytological observations confirmed that the ovules were normal before pollination [4]. This result is similar to that of the GA_4+7_ treatment, which suggests that the ovule death of ‘1913’ is triggered by external signals. Taken together, GA appears to activate different metabolic processes or developmental signaling in seedless and seeded pears, and it triggers the potential PCD process in ‘1913’ ovules.

Next, we analyzed the contents of endogenous hormones in different samples and found that ACC, JA and SA may participate in the PCD process of ‘1913’ ovules (Figure 2). By analyzing these relationships, we determined that JA and ethylene respond to GA_4+7_ treatments, but SA does not (Figure 2), and the expression pattern of their pathway-related genes also indicated their relationship with GA_4+7_ treatment (Figure 3). However, we found that GA_4+7_ treatments significantly increased the JA and ACC levels in the two seeded pear ovules, compared with the control (Figure 2), but the treatments did not cause ovule death as in the ‘1913’ seedless pear. This may result from the positive regulatory roles of GAs on ovule development. Although the JA and ethylene levels increased in seeded pear ovules, their levels were lower than in ‘1913’ ovules. Therefore, they were unable to cause ovule death. Moreover, in the relationship between GAs and JA, ethylene has also been reported. In *Arabidopsis*, GA promotes the expression of the JA biosynthetic gene *DAD1* and JA biosynthesis, and through DELLAs, it regulates the expression levels of *MYB21*/*24*/*57*, which in turn promote stamen development [27]. In addition, GA activates the degradation of DELLAs, which allows JAZ1 to bind MYC2 and suppress the MYC2-dependent JA-signaling output [28]. Furthermore, *FsACO1* expression is drastically increased in seeds after a GA_3_ treatment, but it is decreased by paclobutrazol, a GA biosynthetic inhibitor [29]. However, there are no clear reports regarding the GA-associated mechanism that regulates ethylene biosynthesis.

### 3.2. JA, SA and Ethylene Involvement in Pear Ovule PCD 

Different types of PCD play crucial roles in plant reproductive and vegetative development, as well as in responses to environmental stresses. In this study, we found that a GA_4+7_ treatment triggered potential PCD in ‘1913’ ovules (Figure 1). Further analyses determined that JA, SA and ethylene were present at higher levels in ‘1913’ than in seeded pears (Figure 2), and that applying JA, SA or ACC to normal ‘Bartlett’ ovules resulted in the promotion of ovule PCD (Figure 5).

The lipid-derived stress hormone JA regulates plant adaptations to biotic stresses [30], and it also regulates various aspects of development, including stamen and ovule development, root growth, flowering and leaf senescence [31,32,33,34]. In *Arabidopsis*, *myc3* and *myc4* plants have larger ovules and longer outer integuments than wild-type plants at 0 days after pollination, and *MYC3/4* restrict cell proliferation and cell elongation in the ovules after fertilization [34]. Thus, JA signaling may regulate ovule development. In addition, JA regulates leaf senescence in a variety of plant species [35,36,37,38]. In *Arabidopsis*, MYC2 directly triggers the expression of *SAG29* to regulate leaf senescence [8]. Moreover, exogenous applications of methyl-JA promote leaf senescence in attached and detached leaves [35]. Thus, JA appears to have a senescence-promoting role. Although there are no clear reports on the functions of JA in ovule senescence, ovules derived from specialized meristematic regions within the carpels, referred to as the placentas, are proposed to have evolved from ancestral foliar organs, either leaf-like sporophylls that folded to enclose the ovules or bract-like structures that subtend shoot-like ovules [17]. This evidence suggests that JA may regulate ovule senescence and have a similar regulatory mechanism to leave senescence. In this study, we found that an exogenous application of JA promoted ovule senescence (Figure 5) and that MYC2 triggered the expression of *PbSAG39*, leading to ovule senescence (Figure 6).

In plants, ethylene, JA, auxin and strigolactones have been implicated in development-controlled PCD signaling [8,9,39]. Among them, ethylene is the best-characterized dPCD phytohormone [7]. In *Arabidopsis*, fertilization triggers the ethylene signaling that contributes to the elimination of the persistent synergids via cell fusion and nuclear degradation, which terminates pollen tube attraction [11,40]. In *Zinnia elegans*, the chemical inhibition of ethylene signaling delays xylem differentiation but directly blocks PCD [41]. Ethylene is also involved in pistil fate by modulating the onset of ovule senescence [14]. In our previous study, we reported that ethylene over-accumulates in ‘1913’ ovules after pollination and that the ethylene signaling acceptor PbEIL1 directly activates the expression of *PbCysp1* [4]. Thus, we have determined that ethylene participates in the PCD process of ‘1913’ ovules and that ethylene controls downstream PCD execution. Thus, we have determined that ethylene participates in the GA_4+7_-induced PCD process of ‘1913’ ovules, but we did not conduct research on how the regulatory mechanism of ethylene affects ‘1913’ ovule abortion in this study.

As the only hormone strictly required for the establishment of pathogen-triggered PCD (pPCD), SA promotes pPCD, leading to susceptibility to necrotrophs and biotrophs [42,43]. In addition, SA regulates flower formation, seed germination, embryo development and senescence. In this study, we found that SA was present at a higher level in seedless pear ovules than in seeded pear ovules, but there was no significant difference in its level between GA_4+7_-treated and control groups (Figure 2 and Figure 3). Royo et al. reported that SA has potential roles in promoting grape seed abortion [44]. Additionally, the SA content is higher in seedless ‘Thompson’ grapes than in seeded grapes, and *HDZ28* positively regulates SA biosynthesis, leading to seed abortion [25]. Thus, SA appears to play roles in the PCD progress and is associated with seed abortion in seeded plants. In our results, although SA did not respond to the GA_4+7_ treatment of ovules (Figure 2 and Figure 3), the exogenous spraying of SA induced the expression of the senescence-related genes *PbCysp1* and *PbSAG39* and increased ‘Bartlett’ seed death (Figure 5). This suggests that SA is involved in ‘1913’ seed abortion. Further studies will be conducted to determine the regulatory mechanism.

## 4. Materials and Methods

### 4.1. Plant Materials and Treatments

The samples used in study were collected from a pear orchard in Meixian, Shaanxi, China, in 2021 (34.29° N, 107.76° E, 514 m above sea level) [45]. The average annual precipitation at this location was 574.6 mm, and the average annual temperature was 12.7 °C. The average temperature was 12 °C during anthesis. 

In this study, seedless cultivar ‘1913’ pear (*Pyrus hybrid*), and seeded cultivars ‘Dangshansu’ pear (*Pyrus bretschneideri* Rehd.) and ‘Bartlett’ (*Pyrus communis* L.), were selected as materials. Flowering of the three varieties occurs in late March. The pear cultivar ‘1913’ is a hybrid selected from a cross of ‘Bartlett’ with a male parent of ‘Zaosu’, a hybrid of ‘Shenbuzhi’ (*Pyrus communis* L.) and ‘Pingguoli’ (*Pyrus bretschneideri* Rehd.). For ‘1913’, it has been reported in our previous study that the block of fertilization cause ovule abortion [4]. The seedless characteristic of ‘1913’ has proven stable over many years of observation. Two days before flowering, all selected materials were bagged to prohibit pollination. Healthy and uniform plants were subjected to two treatments: (i) application of water, containing 0.1% ethanol (*v*/*v*) on unpollinated flowers, serving as the control, and (ii) spraying a solution of 50 mg L^−1^ of GA_4+7_ on unpollinated flowers, where the concentration was determined by Wang et al. [45]. Each treatment consisted of 200 inflorescences. The same treatment was performed in the three pear varieties. Samples were collected at 0, 8 and 16 days after treatment (DAT). Several samples were used for observation of ovule development, which were examined under a microscope (MZ10F, Leica, Wetzlar, Germany). The other samples were collected and the ovules were separated from it quickly, then the ovules samples were divided into two groups, including the complete ovules group and the separated ovules group for different experiments. The remaining samples were immediately frozen in liquid nitrogen and stored at −80 °C for total RNA extraction. 

### 4.2. Fluorescein Diacetate (FDA) and Trypan Blue Staining

For the FDA staining assay, it was performed as described previously, with minor modifications [46]. Briefly, fresh sample was cut into 1 mm in the longitudinal direction, and washed with phosphate buffer saline (PBS) 3 times, then the sample was immersed in FDA working solution: a stock solution of FDA (Coolaber, Beijing, China) was prepared by dissolving 5 mg mL^−1^ in acetone, then the samples were stained for 10 min with 12.5 μg mL^−1^ FDA, which was diluted with deionized water. After the extra dye was removed by washing with deionized water for 5 min and repeating 3 times, the ovule sample was observed under a laser scanning confocal microscope (TCS-SP8 SR, Leica, Wetzlar, Germany). Excitation and emission wavelengths were 485 and 530 nm, respectively.

For trypan blue staining, the method was performed as described previously, with minor modifications [47]. For staining, the samples were immersed in boiled lactophenol (glycerol:lactic acid:liquid phenol:distilled water, 1:1:1:1) with 0.25 mg mL^−1^ of trypan blue for 10 min. Then, the samples were de-stained with 2.5 g mL^−1^ of chloral hydrate aqueous solution for at least 36 h. Next, the sample was observed under a microscope (SZX16, OLYMPUS, Tokyo, Japan).

### 4.3. qRT-PCR Validation of Gene Expression Levels

Total RNA was extracted from the ovule sample by using an RNAprep Pure Plant Kit (Tiangen, Beijing, China). Then, 1 µg of total RNA was reverse-transcribed into cDNA following the manufacturer’s instructions of the PrimeScript RT Reagent Kit with a gDNA Eraser (Takara, Dalian, China). Next, the qRT-PCR assay was used to analyze the expression of all detected genes. Sequence similarities were examined based on *Pyrus bretschneideri* genome data, and gene-specific primers (Supplementary Table S1) were designed using Primer 5 software. Then, RT-PCR and melting curve analysis were used to determine the primer specificity. The qRT-PCR assay was performed on an ABI StepOnePlus Real-Time PCR System (Thermo Fisher Scientific, Waltham, MA, USA) using TB Green Premix Ex Taq II (Tli RNaseH Plus, Takara, Dalian, China). Transcripts of *PbActin7* (LOC103926846) served to standardize the cDNA from our test genes. For each sample, three biological replicates were used in the assay. For data analysis, the relative expression level of each gene was calculated using the cycle threshold (Ct) 2^−ΔΔCt^ method [48].

### 4.4. Phytohormone Detection

Concentrations of endogenous hormones, including GA_4_, aminocyclopropane-1-carboxylic acid (ACC, the precursor of ethylene), JA, SA, IAA, ABA and cytokinins (trans-zeatin and trans-zeatin riboside), in ovules of ‘1913’, ‘Bartlett’ and ‘Dangshansu’, were extracted as described previously [4]. The detailed method is referred to in our previous study [4].

### 4.5. Application of JA, SA and ACC on Ovules In Vitro

Fruit of ‘Bartlett’ were collected at 30 days after pollination, and then ovules were separated and precultured in a Petri dish with PBS-moistened filter paper in the dark. After culturing for 12 h, the ovules with black or brown surfaces were removed, and the rest of the ovules were used for treatments. Water treatment was used as a control, and ovules were also treated with 10 μM ACC [49], 50 μM JA and 250 μM SA [50], respectively. The development of different treated groups was observed, and the ovules were stained at significant difference stages by trypan blue.

### 4.6. Yeast One-Hybrid Assay (Y1H)

The full-length coding sequence of *PbMYC2* was amplified and inserted into the MCS of pGADT7 AD, and the specific truncated promoter sequence fragment of *PbCysp1* and *PbSAG39* was inserted into pAbAi bait vectors. Then, the method of the interaction assay was performed according to Wang et al. [4]. The related primers are listed in Supplementary Table S1.

### 4.7. Dual-Luciferase Assay

The full-length coding sequence of *PbMYC2* was amplified and inserted into the MCS region of a pGreenII 0029 62-SK binary vector (effector vector), and the promoter sequences of *PbCysp1* and *PbSAG39* was inserted into the dual-LUC plasmid pGreenII 0800-LUC (reporter vector). The methods of transformation and result analyses were carried out according to Hellens et al. [51] and Wang et al. [4].

### 4.8. Statistical Analysis

Data were subjected to analysis of variance and tested for significant (* *p* < 0.05, ** *p* < 0.01) treatment differences using Duncan’s test and Student’s *t*-test. The results are presented as mean ± standard deviation (SD) of three replicate samples.

## 5. Conclusions

**Figure 7:**
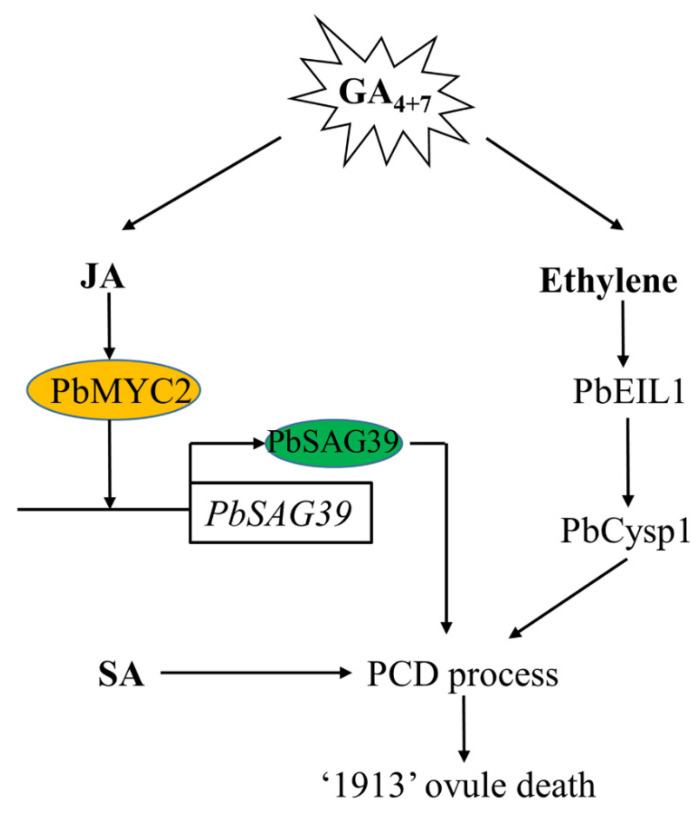
Model depicting the mechanism of GA_4+7_-induced seedless pear ovule abortion in advance. GA_4+7_ treatment increases the production of JA and ethylene, and JA and ethylene signaling receptors PbMYC2 and PbEIL1 directly enhance their target genes *PbSGA39* and *PbCysp1* to regulate pear ovule abortion, respectively. SA also participates in the pear ovule PCD process. GA_4+7,_ Gibberellin A4 and A7; JA, Jasmonic Acid; SA, Salicylic acid; PCD, Programmed Cell Death.

In this study, we demonstrated that GA_4+7_ induces ovule abortion in advance by increasing the endogenous level of JA and ethylene, and SA is also associated with ovule death, though it does not respond to GA_4+7_ treatment. We previously demonstrated that ethylene regulates pear ovule death through PbEIL1, and directly triggers the expression of the ovule senescence-related gene *PbCysp1*, and in this study, we further studied the regulation of JA on ovule death, whereby JA participates in pear death through PbMYC2, binds the promoter of *PbSAG39* and increases its expression, leading to ovule death (Figure 7).

## Data Availability

All data generated and analyzed during this study are included in this published article. The ethics approval and consent to participate are not applicable.

## Supplementary Materials

The following are available online at https://www.mdpi.com/article/10.3390/ijms22189844/s1.
